# Conservation of Indigenous Pig Breeds in Vietnam: Genetic Characterization, Conservation Strategies, and Future Perspectives

**DOI:** 10.3390/biology15090730

**Published:** 2026-05-05

**Authors:** Thanh Van Nguyen, Nguyen Van Ba, Lan Doan Pham, Duy Ngoc Do

**Affiliations:** 1 Faculty of Veterinary Medicine, Viet Nam National University of Agriculture, Hanoi 100000, Vietnam; thanhnv81@vnua.edu.vn; 2 Key Laboratory of Animal Cell Biotechnology, Vietnam Institute of Animal and Veterinary Sciences, Thuongcat, Hanoi 100000, Vietnam; nguyenba81@yahoo.com; 3 Institute of Research and Development, Duy Tan University, Danang 550000, Vietnam; 4 School of Medicine and Pharmacy, Duy Tan University, Danang 550000, Vietnam; 5 Department of Animal Science and Aquaculture, Dalhousie University, Truro, NS B2N 5E3, Canada

**Keywords:** conservation, indigenous breeds, livestock biodiversity, microsatellite, pigs, Vietnam

## Abstract

Indigenous pig breeds in Vietnam represent an important genetic reservoir, offering traits adapted to local environments, cultural preferences, and disease resistance. However, rapid industrialization and the expansion of commercial breeds have endangered many indigenous populations. This review explores the trajectory of conservation efforts for Vietnamese local pig breeds, from the early use of microsatellites and mitochondrial DNA to recent advances in SNP genotyping. Firstly, we summarize the key characteristics and values of 26 local breeds. Secondly, we highlight key findings on genetic diversity, population structure, and inbreeding levels across major breeds. In addition, we discuss challenges in the development of conservation breeding programs and national strategies, as well as challenges in data generation, infrastructure, and policy implementation and provide potential solutions for these challenges. This review provides the first integrated synthesis linking breed-level genetic evidence to practical conservation recommendations for indigenous pigs in Vietnam. By identifying the key breeds for conservation priority, such as Huong, Van Pa, Soc, ChuProng, Co Aluoi, and Lung Pu, as well as highlighting the exotic introgression in H’mong pig populations, this review might provide a resource for sustainable conservation and use of Vietnam’s rich pig genetic resources.

## Simple Summary

Local pig breeds in Vietnam are an important part of the country’s agriculture and culture. They are well adapted to local climates, traditional farming systems, and diseases. However, many local breeds are disappearing because modern commercial pig breeds are being used more widely. This review describes the characteristics of Vietnamese local pig breeds and explains why they are valuable. It also summarizes their genetic diversity based on genetic technologies. Finally, the review discusses problems that limit conservation efforts, such as a lack of information, facilities, and policy support. This information can help protect Vietnam’s local pig breeds for the future.

## 1. Introduction

**Figure 1:**
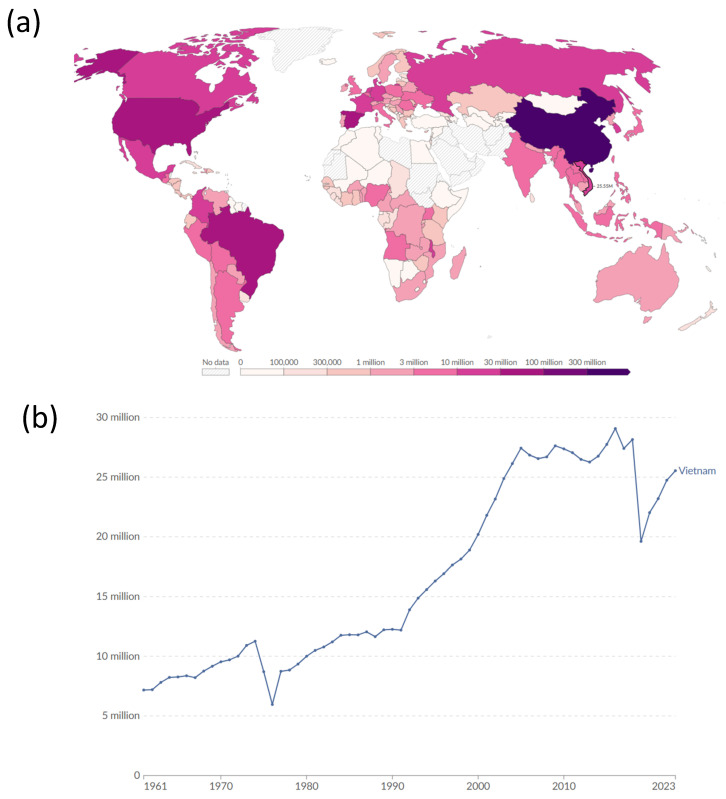
Number of pigs in the world in 2023 (**a**) and in Vietnam (**b**). Source Our World in Data: https://ourworldindata.org/ (accessed on 12 October 2025).

The pig is one of the most important livestock species in Vietnam; it is not only the main meat product for consumers, but also has special cultural values for Vietnamese people [1,2]. In fact, pig production is one of the most important agricultural sectors, as pork accounts for the largest share of meat consumed in the country, placing Vietnam among the world’s top-producing and pork-consuming nations, with an estimated 25.55 million pigs in 2023 [1] (Figure 1). The pig industry is currently in a state of dynamic transition, moving away from its historical foundation of small-scale, traditional farming systems towards large-scale, industrial, and modernized production chains [2]. This transformation, driven by rising domestic demand, global integration, and the necessity for enhanced biosecurity, has significantly impacted the country’s pig population [2,3,4]. While the focus of modern commercial farming relies heavily on high-yield exotic breeds and their crosses, the foundation of the country’s pig legacy lies in its rich heritage of indigenous pig breeds.

Vietnam is home to 26 native pig populations [5,6,7], such as the highly prolific Mong Cai and the unique I pig (also called Vietnamese Pot-bellied), which have evolved over centuries through selective breeding and environmental adaptation. These local breeds are characterized by their exceptional genetic diversity, robust resistance to endemic diseases, and remarkable adaptation to harsh, low-input environments, particularly in mountainous and remote regions [5,8]. Furthermore, they also have a higher price for meat compared to the commercial breeds because of their superior taste and local preferences [9,10,11,12]. However, the rapid push toward industrial efficiency has caused significant genetic erosion, with many native breeds facing the risk of extinction [11,13]. Understanding the diverse genetic and phenotypic characteristics of these native Vietnamese pigs is therefore critical for conservation prioritization, breeding program design and evidence-based conservation policy development [13,14,15]. Therefore, a summary of the diversity of Vietnam’s swine genetic resources, the characteristics and ecological adaptations of its key local breeds facing challenges, and perspectives on local breed conservation is vital. This review is organized around four objectives: (i) to summarize the phenotypic and productive characteristics of 26 Vietnamese indigenous pig breeds; (ii) to synthesize findings on genetic diversity, population structure, and inbreeding from microsatellite, mtDNA (complete mitogenome sequences obtained by Sanger sequencing), and SNP marker studies; (iii) to evaluate in situ, ex situ, and genomic conservation strategies in the Vietnamese context; and (iv) to identify current challenges and future priorities for sustainable conservation of these genetic resources.

## 2. Phenotypic Characteristics and the Importance of Vietnamese Local Pig Breeds

**Figure 2:**
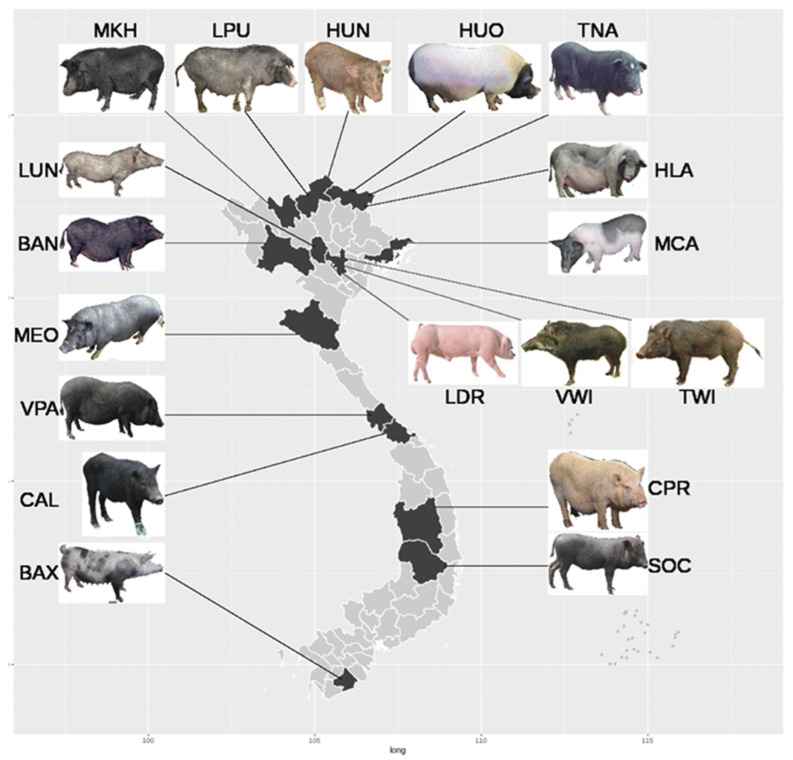
Major indigenous pig breeds in Vietnam: Ha Lang (HLA), Huong (HUO), Tap Na (TNA), Hung (HUN), Lung (LUN), Muong Khuong (MKH), Meo (MEO), Lung Phu (LPU), Mong Cai (MCA), Ban (BAN), ChuProng (CPR), Van Pa (VPA), Soc (SOC), Co Aluoi (CAL), and Ba Xuyen (BAX). Adapted from Ba et al. [6].

Indigenous Vietnamese pig breeds represent a vital component of the country’s agricultural biodiversity. These breeds have evolved over centuries through adaptation to diverse local environments, including mountainous regions, coastal areas, and the Red River and Mekong Deltas (Figure 2). They are characterized by resilience to harsh conditions, disease resistance, and suitability for smallholder farming systems and also have great potential economic values (Table 1) [16]. Furthermore, indigenous Vietnamese pigs exhibit remarkable disease resistance shaped by long-term adaptation to tropical conditions. For example, Ban pigs maintained under free-range, high-exposure systems in Hoa Binh showed only moderate seroprevalence of trichinellosis (13.6%) and very low prevalence of T. solium cysticercosis (1.7%), indicating resilience to parasitic infections despite risky husbandry practices [17]. Similarly, Mong Cai pigs display stronger humoral and cell-mediated immune responses compared to exotic Yorkshire and Landrace breeds under the same management, conferring superior resistance to common infectious diseases, while exotic breeds show greater susceptibility to environmental stressors and pathogens [18]. Phenotypically, many breeds exhibit compact bodies, varied coat colors (predominantly black or black and white), and traits suited to free-range or semi-intensive rearing. Their values extend beyond production, encompassing economic importance for rural ethnic minority communities, cultural significance, and genetic resources for conservation and potential biomedical applications. The summary of economic contribution is listed in Table 2. However, many face extinction risks from crossbreeding with exotic breeds, leading to genetic erosion [19]. Conservation efforts, including relational databases and assisted reproductive techniques, aim to preserve their diversity. Below, we detail the phenotypic appearances and values of the major breeds, focusing on key traits like coat color, body shape, reproductive performance, and socio-economic roles [20,21]. Breeds are grouped by regional clusters where applicable, with populations sharing names treated as variants [22].

In northern and northwestern Vietnam, breeds exhibit adaptations to rugged terrains, including hardy constitutions, high fat deposition for energy reserves, and strong prolificacy, making them integral to ethnic groups. Mong Cai is one of the most economically important indigenous breeds in Vietnam, originating from the northeastern coastal provinces. This breed exhibits distinctive black and white coloration, with black patches typically covering the head and rump while the middle portion remains white. Adults are medium-sized, with mature boars weighing 80–120 kg and sows weighing 70–100 kg [23,24]. The breed is characterized by a broad forehead, short snout, large drooping ears, and a slightly concave dorsal profile. It also has a superior meat quality based on local preference, with high intramuscular fat content (3–5%) contributing to tenderness and flavor. Mong Cai pigs possess exceptional prolificacy, with litter sizes averaging 12–14 piglets, and can reach up to 18 piglets per litter, making them highly valued for crossbreeding programs. They demonstrate excellent mothering ability and early sexual maturity (4–5 months) [23,24].

Like Mong cai pigs, I pig breeds are distributed throughout the northern regions of Vietnam [25]. However, they are considered as small-framed animals, with adult boars weighing 50–70 kg and sows 45–60 kg [23]. This breed displays uniform black coloration across the body, though some individuals may have small white markings on the feet or the tip of the tail. Morphologically, I pigs feature a straight or slightly dished face, medium-sized erect ears, and a compact body conformation. The breed is renowned for its extreme hardiness and ability to forage efficiently in mountainous terrain with minimal supplementation. I pigs produce high-quality meat with excellent marbling (4–6% intramuscular fat) and are particularly valued in local markets for their superior taste [23]. Their heat tolerance and disease resistance make them ideal for low-input farming systems in remote areas.

The Ban breed predominantly features black coats (with some black-and-white variants in Bac Kan and Yen Bai provinces), plain patterns, average hair density often with a mane (56–62%), black skin, straight faces and snouts, horizontal ears, slim or drooping bellies, and straight or swayback backlines [26,27]. This breed is raised primarily by ethnic minority communities and has an average of 10–11 teats, with adults at 50–80 kg [26,27]. They thrive in upland free-range systems, offering genetic diversity and cultural importance, but admixture with exotics threatens purity. This breed is highly valued for its ability to survive and produce under harsh conditions with minimal inputs. They efficiently utilize forest resources during foraging and demonstrate excellent disease resistance. The meat quality is exceptional, with high intramuscular fat content (4–6%) and distinctive flavor profiles that reflect their natural diet, making them popular for traditional fermented pork products [26,27].

Other breeds in northern Vietnam noted for their ability to adapt to low production systems include Muong Khuong, Hang Lang, Huong, Tap Na, Lan and Muong Lay. The Muong Lay breed from Dien Bien province is traditionally raised free-range. Most females have 14 teats, some even 16 and can birth up to 20 piglets. Though once common among Thai and Hmong communities, their numbers have declined. Muong Lay pigs are hardy, thrive on low-nutrition feed, resist disease, and produce tasty meat. They are typically raised in small household settings, with families breeding their own stock and borrowing boars locally [28]. The Muong Khuong breed in Lao Cai displays a black coat, downward ears, average hair, sometimes with white markings on the feet and face, 10–11 teats, and larger adult sizes (130–150 kg) [29]. This breed possesses a compact body structure, short legs, a slightly dished face, and small, erect ears [23]. They have a small litter size (4–5 pigs/litter) [30]. Similar to the Mong Cai and I pig breeds, Muong Khuong is known for great meat quality characterized by fine muscle fibers and high intramuscular fat content (4–5%), making it highly desirable for traditional Vietnamese cuisine. This breed is exceptionally adapted to cold mountainous climates and demonstrates remarkable foraging ability on natural vegetation and crop residues [31]. Muong Khuong pigs are valued for their low maintenance requirements and suitability for smallholder farming systems in harsh environments [23,32,33]. Ha Lang and Huong pigs in Cao Bang province share black-and-white coats, black on the head and buttocks with a predominantly white body, along with swayback conformation and drooping bellies. Both breeds typically have around 12 teats and show high reproductive efficiency. Ha Lang is a relatively larger-framed breed, with mature boars reaching approximately 100 kg, and is noted for its high teat number (12–14), which is associated with superior litter-rearing capacity [5,23]. Tap Na pigs are predominantly black, occasionally with white spots on the head, tail, feet, and belly. They have a concave facial profile, pendulous ears, and an elongated trunk, and are classified as a small-bodied breed [34]. Both Ha Lang and Tap Na are well adapted to low-input extensive systems in the northeastern uplands. Lan pigs are small in body size (boars 40–65 kg; sows 35–55 kg), compact, frequently piebald, with erect ears and pronounced ham musculature; they exhibit early maturity and 4–5 cm backfat [14,35]. Reportedly, piglets are primarily retained for home consumption rather than commercial use [23].

Central Vietnam hosts fewer indigenous pig breeds compared to the northern regions. The Co breed complex comprises five geographically distinct subpopulations in Thanh Hoa, Quang Nam, Gia Lai, Binh Thuan, and Thua Thien Hue provinces. They are all predominantly black (92–98% solid coat), with plain bristle patterns, dorsal hair manes in 38–57% of individuals, black skin, straight rhinarium, horizontal ears, concave dorsal line, and slim ventral profile [23]. Teat counts average 10–12, the lowest recorded among indigenous Vietnamese pigs; adult live weight ranges 32–58 kg, with backfat thickness 3.8–5.2 cm at 70 kg [23]. These breeds all excel in gastrointestinal parasite resilience and heat tolerance under coastal and mid-altitude extensive systems. Khua pigs are an indigenous breed raised by the Khua ethnic group in Minh Hoa district, Quảng Bình province. Khua pigs forage on their own and are mainly found in remote border communes. Due to declining numbers, this breed is at risk of disappearing, representing a loss of valuable local and national genetic resources [23].

There are a few indigenous breeds (ChuProng, Soc, O Lam and Ba Xuyen) in the southern and highland regions of Vietnam. ChuProng in the Gia Lai highlands has mostly black coats (88%), white skin elements, low spotted patterns, straight bodies, and 10–11 teats. Soc in Dak Lak resembles northern types with black coats, straight backlines, slim bellies, and 10–11 teats, valued for highland resilience. The O Lam breed in An Giang province (Mekong Delta) features white or black–white coats and skin (35–60%), spotted patterns, and high teat variation (12.9 ± 1.3). The Ba Xuyen breed is a derived composite breed specific to southern Vietnam, particularly the Mekong Delta region. This breed was developed through the crossbreeding of Berkshire pigs (imported 1932–1958) with the Bo Xu breed (composite of Craonnais pigs and Chinese pigs) [23]. It was listed as extinct by the National Institute of Animal Science (NIAS) in 2015.

## 3. Genetic Diversity of Local Breeds

Several types of molecular markers (microsatellites, mtRNAs and SNPs) have been used for characterizing the genetic diversity of indigenous pig breeds in Vietnam. Most of the methods showed a high level of genetic diversity for local breeds.

### 3.1. Genetic Diversity of Vietnamese Indigenous Pigs Based on Microsatellites

Microsatellite markers have been widely used as genetic tools to assess genetic diversity in Vietnamese pig breeds. Thuy et al. [14] compared Vietnamese breeds (Mong Cai, Muong Khuong, Co, I, Meo, and Tap Na) with European breeds using 20 microsatellite markers, demonstrating that Vietnamese breeds maintained higher allelic diversity despite smaller population sizes, but showed evidence of genetic admixture with exotic breeds. Pham et al. [7] analyzed five indigenous populations (Lung Pu, Ha Lang, Muong Te, Lung, and Mong Cai) using 16 microsatellite markers across a total of 241 samples and found remarkably high genetic diversity across all breeds. The authors found that the Lung Pu breed exhibited the highest diversity indices with a mean number of alleles of 10.1, expected heterozygosity (He) of 0.82, and allelic richness of 5.33, and the overall mean He across Vietnamese breeds was significantly higher than many commercial European breeds. Berthouly-Salazar et al. [13] focused on the genetic status (genetic diversity and introgression) from exotic breeds of the Vietnamese Black H’mong pig breed in Ha Giang province. The authors analyzed three phenotypes, Black, Spotted, and White, and found low genetic differentiation among them (0.5–6.1%). The White phenotype showed intermediate admixture values, suggesting that it is a crossbreed between local and exotic pigs (Landrace and Yorkshire). In addition, the authors performed modeling based on local management practices, which predicts that 100% of Black H’mong private alleles will be lost within 60 generations due to ongoing introgression. The study highlights the difficulty of identifying hybrid classes in closely related populations. Ba et al. [6] analyzed 15 breeds using 19 microsatellite markers and reported varying diversity levels among local breeds. The authors revealed that approximately 15% of genetic variation occurred between breeds, indicating substantial population differentiation. In the most comprehensive studies using microsatellites in local pig breeds in Vietnam, Ba et al. [5] characterized 24 Vietnamese pig breeds using 20 ISAG/FAO-recommended microsatellite markers across 1136 samples. The authors revealed an average of 10.0 alleles per locus and an allelic richness of 7.6, confirming the exceptional genetic diversity maintained in Vietnamese pig populations. These values exceeded those typically observed in exotic commercial breeds [5]. Although the cited studies employed overlapping but non-identical microsatellite panels (20 loci in Thuy et al. 2006 [14], 16 loci in Pham et al. 2014 [7] and Berthouly-Salazar et al. 2012, and 19 loci in Ba et al. 2020 [5], sharing at least 12–15 FAO/ISAG core markers including SW2410, SW936, S0068, SW857, S0097, SW240, SW72, SW1067, S0215, S0225, S0227, SW122, SW632, S0355, and S0226), they enable the direct comparison of diversity metrics, yet reveal contrasting patterns. Thuy et al. (2006) [14] and Pham et al. (2014) [7] emphasized high genetic purity and diversity in northern indigenous breeds, reporting an elevated mean number of alleles (MNA 8.1–10.1), expected heterozygosity (He up to 0.82 in Lung Pu), and numerous private alleles, with clear separation from exotic breeds in neighbor-joining trees and Bayesian clustering. In contrast, Berthouly-Salazar et al. (2012) [13] documented rapid genetic erosion via uncontrolled admixture in H’mong pigs from Ha Giang, where White phenotypes represent first-generation hybrids (admixture coefficient q = 0.53 with Landrace/Yorkshire) and Spotted phenotypes reflect multi-generational crossing, projecting complete loss of Black H’mong private alleles within 60 generations under current admixture rates (AR = 0.25) and mortality (MR = 0.4). Ba et al. (2020) [6] bridges these views by identifying 15% among-breed variation and 12 DAPC clusters across 15 indigenous breeds. The authors confirmed high authenticity in Huong, Van Pa, Soc, ChuProng, and Co Aluoi (which have distinct clusters with low bootstrap support for admixture) while flagging Ba Xuyen as highly susceptible to erosion (as it clusters tightly with Landrace; q > 0.95 exotic ancestry). Thus, breeds most susceptible to genetic erosion include H’mong phenotypes (Black/Spotted/White) and Ba Xuyen, whereas Huong, Van Pa, Soc, ChuProng, Co Aluoi, and northern isolates such as Lung Pu have retained the highest authenticity and warrant elevated conservation priority.

### 3.2. Genetic Diversity of Vietnamese Indigenous Pigs Based on Mitochondrial DNA

Mitochondrial DNA studies have provided valuable insights into the maternal lineages and evolutionary history of Vietnamese pigs. The D-loop region, typically 574–713 bp in length, has been the primary target for mtDNA sequencing studies. Hongo et al. [36] identified 50 distinct haplotypes in Vietnamese pig populations, including 27 novel haplotypes not previously reported in other Asian pig populations [36]. These haplotypes were classified into two major haplogroups, the Mainland South-East Asian (MTSEA) group and the D2 group, with the MTSEA lineage being predominant in most Vietnamese breeds. In an analysis of the 16,742 bp mtDNA sequence of the Muong Lay breed, Vo et al. [27] indicated its position within the East Asian maternal lineage. The breed shares close genetic affinities with Chinese pigs from the Yangtze and Yellow River basins but remains distinct from European stocks. Beyond its evolutionary placement, the breed is characterized by a strong A + T bias and a D-loop containing 17 repeat sequences, a feature the authors suggest may drive specific replication errors and shape the breed’s genetic landscape. Nguyen et al. [37] sequenced the 16,731 bp mitochondrial genome of the Vietnamese I pig and revealed a typical porcine mitogenome structure with 37 genes and 60.09% A + T content in the mitogenome. The authors confirmed that the I pig was phylogenetically placed in a distinct Asian clade with the Banna mini pig and Vietnamese wild boar. Thuy et al. [29] analyzed mtDNA diversity among six Vietnamese local pig breeds, namely, I, Mong Cai, Muong Lay, Muong Khuong, Hạ Lang, and Huong, by sequencing the complete mitochondrial genomes. The authors indicated that the D-loop region showed 25 polymorphic sites, indicating moderate genetic variation, with I pig exhibiting the highest number of mutations among the six breeds. Using phylogenetic analysis of 33 global pig breeds, the authors revealed that all Vietnamese native pigs cluster within the Asian lineage and share maternal ancestry closest to Ryukyu wild boars of Japan, suggesting ancient genetic continuity in Southeast Asian pigs.

### 3.3. Genetic Diversity of Vietnamese Indigenous Pigs Based on SNPs

Single-nucleotide polymorphism (SNP) analysis has become a foundation in understanding the genetic diversity in many parts of the world, with advances in SNP panel development and genome sequencing technology. However, the majority of SNP applications in indigenous pigs in Vietnam are based on conventional genotyping methods, such as PCR or PCR-RFLP, with analysis limited to a small number of candidate SNPs or genes. For instance, Cuong et al. [38] identified 17 SNPs in the H-FABP gene, revealing polymorphisms associated with intramuscular fat and meat quality. The authors emphasized the low frequency of favorable alleles in native breeds, with Tap Na pigs showing high resistance and fat content. The first application of SNP chips in the Vietnamese breed was performed by Ishihara et al. [15], who conducted a comprehensive study using a 60K SNP chip across 15 Vietnamese pig breeds. Based on F-statistics (F_ST_) estimates, the authors indicated moderate-to-high differentiation between geographic clusters, with northern breeds (Mong Cai, Ha Lang, and Muong Khuong) forming distinct genomic groups from southern breeds [15]. The analysis confirmed high genetic diversity within breeds while also detecting varying degrees of admixture with exotic commercial breeds in some populations. The authors also indicated that, despite some introgression from exotic breeds, Vietnamese pigs maintain distinct genomic signatures that differentiate them from European and other Asian pig breeds [15]. However, functional variants associated with disease resistance, heat tolerance, and feed efficiency, such as traits that are central to the conservation value of these breeds, remain largely uncharacterized at the genomic level and represent a priority for future population genomic studies.

Across all three marker systems, a consistent picture emerges of high within-breed genetic diversity in Vietnamese indigenous pigs, generally exceeding levels observed in commercial European breeds. Microsatellite and SNP data agree in identifying geographic clustering as the primary driver of population structure, with northern, central, and southern breeds forming broadly distinct genetic groups. Mitochondrial DNA analysis further confirms that all Vietnamese breeds share Asian maternal lineages, clustering within the East/Southeast Asian clade. However, some discordance is apparent at the breed level: breeds such as the Black H’mong pig show evidence of exotic admixture in microsatellite data that is not always captured by D-loop haplotyping alone, underscoring the value of multi-marker approaches for conservation decision-making. The scarcity of genome-wide SNP data for Vietnamese indigenous pigs is due to the cost of 60K SNP arrays relative to typical project budgets in Vietnam and the difficulty of assembling sample sizes sufficient for robust population genomic inference across breeds with only a few hundred registered individuals. These gaps mean that functional variants underlying the adaptive traits central to the conservation value of these breeds’ disease resistance, heat tolerance, and feed efficiency remain entirely uncharacterized at the genomic level. In addition, the absence of whole-genome sequencing data for most breeds represents a gap in population genomics, which should be prioritized in future studies.

## 4. Conservation Strategies for Indigenous Pigs in Vietnam

The integration of genomic technologies into conservation and breeding programs represents a critical pathway for the sustainable management of Vietnamese pig genetic resources. Traditional conservation approaches have focused primarily on maintaining population numbers through in situ management in conservation farms, but molecular data now enable more sophisticated strategies that balance genetic diversity preservation with genetic improvement. The establishment of national conservation frameworks should incorporate genomic information at multiple levels, from population structure analysis to individual mating decisions, ensuring that breeding programs simultaneously maintain genetic diversity while achieving modest genetic gains in economically important traits. Vietnam has undertaken various efforts to conserve its indigenous pig breeds, supported by both national programs and international collaborations. Conservation strategies can be broadly categorized into in situ (on-farm) and ex situ (off-farm) approaches. In situ conservation aims to maintain breeds within their natural production environments, allowing them to continue adapting and evolving [39]. This method supports cultural preservation and engages local communities directly in conservation activities. Ex situ strategies include the cryopreservation of semen and embryos, as well as the establishment of conservation herds in research institutions and government farms [40].

### 4.1. In Situ Conservation and Community-Based Conservation

Community-based conservation approaches directly involve farmers and local organizations in the management and breeding of indigenous pig populations. In Vietnam, pig farming, especially among ethnic minorities in mountainous regions, is a primary livelihood activity for many rural households [31], which might facilitate the applications of community-based conservation. Indigenous breeds like the Ban, Mong Cai, and I pigs are well adapted to local environments, provide high-quality meat preferred by consumers, and have low input requirements, making them suitable for resource-limited farmers [31]. By empowering communities to steward their own animal genetic resources, these programs create a sense of ownership and responsibility. This involvement is vital for maintaining breed purity, preserving unique genetic traits, and generating opportunities to improve household income via specialty products and local niche markets. Community breeding associations further support breed conservation and act as knowledge-sharing platforms, linking traditional practices with new breeding technologies [41,42]. In Son La province, the Ban pig community breeding association established with support from NIAS has provided a practical model, implementing boar rotation protocols, and demonstrating stable maintenance of allelic richness over two breeding cycles [43,44]. Additionally, indigenous pig rearing supports women’s participation in household economies since women are often primary caregivers of small livestock in rural Vietnam. Most community-based conservation studies in Vietnam have been conducted in the northwest, primarily involving breeds such as Ban, I, and Mong Cai pigs. The applicability of these findings to southern and highland breeds, such as ChuProng, Ba Xuyen, and Soc, requires careful consideration, as the socio-economic contexts, cultural relationships with livestock, market integration levels, and land-use systems differ substantially across Vietnam’s regions. Future conservation programs should incorporate regionally tailored approaches developed in collaboration with local communities.

### 4.2. Ex Situ Conservation and Gene Bank Development

The development of genome resource banks (GRBs) provides cost-effective long-term conservation solutions while facilitating genetic exchange and supporting assisted reproductive technologies. For local Vietnamese pig conservation, a tiered GRB system should be established following international best practices: a National Reserve containing germplasm from all recognized breeds preserved for worst-case scenario reconstruction; a Savings Account with strategic samples to periodically invigorate genetically depauperate populations; and a Checking Account with regularly used materials for routine breeding program operations [45]. The development of Vietnamese pig GRBs faces species-specific technical challenges, as pig embryos are notoriously difficult to cryopreserve due to their high lipid content and chilling sensitivity, though recent advances in vitrification protocols and in vitro embryo production techniques are improving success rates [46]. Although investment in GRB infrastructure has been estimated to be substantially more cost-effective than maintaining equivalent genetic diversity through live animal populations [47], it may not directly apply to Vietnamese indigenous pig breeds without species- and context-specific economic modeling [47]. Priority should be given to collecting and banking germplasm from genetically unique individuals identified through molecular analyses, particularly males with rare haplotypes or high genetic diversity indices, and from breeds showing evidence of declining population sizes or genetic erosion.

Optimum contribution selection (OCS) has emerged as a powerful tool for managing genetic resources in local breeds, offering a strategic balance between genetic gain and diversity conservation that traditional truncation selection cannot achieve [47]. For Vietnamese local pig breeds with documented exotic breed introgression, advanced OCS approaches that account for the breed origin of alleles and migrant contributions are particularly relevant. These methods enable breeding programs to simultaneously maximize genetic gain, control inbreeding rates at native alleles, and maintain genetic originality, objectives that are inherently conflicting in traditional selection schemes [48,49]. Implementation of genomic OCS in Vietnam would require the establishment of genomic reference populations, the development of breed-specific breeding values for economically important traits such as meat quality and prolificacy, and the training of personnel in advanced selection methodologies. The relatively small population sizes of many Vietnamese breeds, ranging from a few hundred to several thousand breeding individuals, make them particularly suitable for OCS implementation [5]. A simulation study showed that even populations with 300 female reproducers can achieve genetic progress comparable to truncation selection while maintaining inbreeding rates below 1% per generation [50]. However, breed-specific simulation studies are needed to determine appropriate threshold values for Vietnamese indigenous pig breeds, which differ in population size, structure, and level of exotic admixture.

Porcine-specific assisted reproductive technologies (ARTs) present well-known technical challenges, particularly for embryo cryopreservation due to the high lipid content and chilling sensitivity of pig oocytes and embryos. However, recent advances in sperm cryopreservation protocols, including the use of antioxidant-supplemented extenders and optimized freezing curves, have improved post-thaw viability and fertility outcomes for several Vietnamese indigenous breeds. Somatic cell nuclear transfer (SCNT) has also been explored as a tool for preserving the genomes of genetically unique individuals, though its application remains at an experimental stage in Vietnam [51].

## 5. Challenges, Key Elements, and Future Perspectives

### 5.1. Major Challenges for Conservation of Local Pig Breeds

Vietnam has lost at least one indigenous pig breed entirely, as Ba Xuyen is now listed as extinct, and more than 20 of the remaining breeds are classified as at-risk or endangered according to the FAO DAD-IS database [23]. These losses are the result of many factors, including the result of market forces, policy failures, infrastructure gaps, and structural disadvantages that have made it difficult for smallholder farmers to maintain local breeds in the face of competition from exotic commercial lines [52,53,54,55]. Many indigenous pig breeds are deeply tied to ethnic minority livelihoods and cultural traditions, and their survival is threatened by several factors. Firstly, a rapid expansion of commercial pig farming and crossbreeding with exotic breeds has reduced genetic diversity, making local pigs less competitive in terms of growth rate and meat yield [56]. Secondly, smallholder farmers in mountainous regions or rural areas often lack resources and veterinary support, leading to poor herd management and vulnerability to diseases, especially some devastating diseases recently, such as African Swine Fever Virus infection [57]. Thirdly, market demand favors fast-growing commercial pigs, pushing indigenous breeds into niche or subsistence roles rather than mainstream production [12]. In addition, inappropriate policies in certain periods, encouraging importing exotic breeds and ignoring the importance of diversity, have reduced genetic resources. For example, subsidies and import incentives favoring commercial exotic breeds during the 1990s and 2000s accelerated crossbreeding without adequate safeguards for indigenous breed conservation [19]. Finally, rapid industrialization requires more land for industry, which limits habitats for local breeds.

### 5.2. Key Elements in Conservation of Indigenous Pigs in Vietnam

#### 5.2.1. Government and Policy

Policy and institutional frameworks play a pivotal role in the conservation of local breeds, particularly in contexts such as Vietnamese indigenous pigs. Similar to other developing countries, conservation efforts for local breeds in Vietnam are challenged by fragmented populations and weak institutional coordination. Central to these efforts is the Law on Livestock Production (No. 32/2018/QH14), which took effect in 2020 and includes specific provisions for the preservation of indigenous livestock breeds (https://faolex.fao.org/docs/pdf/vie209580.pdf, 2 April 2026). Under this law, the state is responsible for identifying, cataloging, and supporting the conservation of valuable native genetic resources. Complementing the law, the National Strategy for Livestock Development (2021–2030, vision to 2045) outlines goals for sustainable livestock production, which include improving genetic quality while protecting biodiversity. The conservation of local breeds is framed not only as a scientific priority but also as a measure for rural development and food security. Robust government policy forms the foundation for sustainable conservation, enabling the establishment of legal protections that safeguard genetic diversity and secure unique local traits [58]. Targeted subsidies and incentives encourage breeder participation, while education and extension services promote the adoption of best practices and the dissemination of essential technical knowledge among rural communities [59]. Community-based initiatives, empowered by these supportive measures, allow local breeders to manage animal genetic resources, fostering both breed purity and innovation through collective action. However, several challenges hinder effective policy implementation. These include limited coordination between national and local authorities, insufficient funding, and weak enforcement mechanisms. Many conservation programs rely on short-term project funding rather than sustained support. Furthermore, farmers often receive little to no economic incentive to maintain native breeds, especially when exotic crosses offer better short-term productivity. This highlights the need for policy instruments that integrate conservation with market-based incentives, such as eco-labeling, geographical indication certification for native breed products, and community-based breeding cooperatives. The Ministry of Agriculture and Rural Development (MARD) is assigned to lead the development of breed conservation strategies and maintain national breed registries (Table 3).

#### 5.2.2. Local Farmers and Ethnic Groups

Local farmers and ethnic communities constitute pivotal stakeholders in the ex situ and in situ conservation of Vietnamese indigenous pig breeds, leveraging indigenous knowledge systems to sustain genetic diversity through traditional husbandry practices, cultural transmission, and adaptive management. These groups maintain unique adaptive traits, such as resilience to local diseases, foraging efficiency, and environmental adaptability via on-farm rearing, selective breeding based on phenotypic selection, and integration with agroforestry systems in mountainous regions. Their engagement in national initiatives, including the National Program on Conservation of Vietnamese Animal Genetic Resources coordinated by the National Institute of Animal Science (NIAS), involves contractual nucleus herd management, performance recording, and community-based breeding programs that have stabilized populations of breeds like Ban, I and Mong Cai while mitigating inbreeding (http://pigtrop.cirad.fr/subjects/genetic_and_biodiversity/conservation_of_autochthonous_pig_breeds, accessed 10 March 2026). Alongside these efforts, practical capacity building, such as training in biosecurity, trait recording, and market-focused production, helps improve productivity while conserving the genetic integrity of local breeds. Market linkages for niche products provide economic incentives for raising local breeds. Inclusive strategies prioritizing women and ethnic minorities will ensure equitable benefits and long-term stewardship amid climate variability and genetic erosion pressures.

#### 5.2.3. Research and Education Institutions

The conservation of animal genetic resources in Vietnam is supported by several national policies, legal instruments, and institutional programs. Government institutions such as the NIAS and provincial agricultural research centers implement conservation and breeding programs, both in situ and ex situ. The agricultural universities, such as Vietnam National University of Agriculture, play a central role in conservation by combining research, education, and community engagement to protect natural resources and biodiversity. Their contributions span sustainable land management, climate-resilient agriculture, and ecosystem protection, and they work directly with farmers and local authorities to implement conservation practices. They are also actively involved in the conservation of pig breeds as well as training scientists for the field.

#### 5.2.4. International Networks and Collaborations

Vietnam has joined several international agreements, including the FAO Global Plan of Action for Animal Genetic Resources, and has participated in regional initiatives under the Asia–Pacific Regional Strategy for Animal Genetic Resources to conserve local breeds (https://openknowledge.fao.org/handle/20.500.14283/cb3087en, accessed 20 March 2026). Many international collaborations and funding supports have been placed in recent years to support the research and implementation of conservation of pig breeds (Table 3). For example, the genetic analysis of native Vietnamese pig breeds was carried out within a large international research effort under the SATREPS project (https://www.jst.go.jp/global/english/kadai/h2604_vietnam.html, accessed 20 March 2026), which focused on establishing a cryo-bank system to conserve Vietnam’s indigenous pig resources. Supported by the Japan International Cooperation Agency and the Japan Science and Technology Agency, the project brought together universities, research institutes, and private partners from both Vietnam and Japan to build a gene bank for long-term conservation, beginning with a detailed investigation of the genetic characteristics of these native pigs. For example, the project has documented external and morphological traits, such as coat color, to create a comprehensive database of Vietnamese native pig diversity. The project resulted in the comprehensive phenotypic and genetic characterization of 24 Vietnamese indigenous pig breeds across 1136 sampled individuals. The project established a foundational cryo-bank containing semen samples from genetically unique males across multiple breeds, contributing to long-term ex situ conservation. While alignment with global guidelines exists in principle, greater effort is required to ensure these commitments translate into practical, locally tailored actions. Overall, a more cohesive policy framework that integrates genomic tools, local stakeholder participation, and long-term funding is essential to safeguard Vietnam’s unique pig genetic resources.

### 5.3. Future Perspectives

Climate change represents an increasingly critical but underacknowledged threat to Vietnamese indigenous pig breeds and the communities that steward them. Upland ethnic minority communities in the northwest and central highlands, who maintain the largest populations of many rare indigenous breeds, are among the most vulnerable to climate-related disruptions, including shifting rainfall patterns, increased frequency of extreme weather events, and changes in the availability of traditional foraging resources. These pressures may force changes in husbandry practices, increase disease incidence, and reduce smallholders’ capacity to maintain indigenous breed populations.

Several emerging technologies hold promise for the conservation and utilization of Vietnamese indigenous pig breeds. Long-read sequencing platforms (e.g., PacBio and Oxford Nanopore) enable high-quality genome assembly and the detection of structural variants that short-read SNP chips cannot capture, offering deeper insights into breed-specific adaptations. Pangenomic approaches that aggregate reference genomes from multiple individuals will better represent the true genetic diversity of these populations [60]. CRISPR-based tools may facilitate breed characterization by enabling precise functional studies of candidate genes underlying local adaptation traits such as disease resistance and heat tolerance [61]. One Health frameworks offer an integrative lens for understanding the interconnections between indigenous pig conservation, rural livelihoods, and zoonotic disease risk. Finally, the systematic documentation and integration of traditional ecological knowledge held by ethnic minority communities represent both an ethical priority and a practical resource for guiding conservation decisions.

## 6. Conclusions

Growing studies in microsatellites, mtDNA, SNPs, and Sanger sequencing of mitochondrial D-loop regions have documented genetic diversity in Vietnamese indigenous pig breeds, with diversity levels consistently exceeding those of commercial exotic lines. These studies also provide complementary insights into population structure, evolutionary history, and varying degrees of admixture across breeds. The current literature suggests that Huong, Van Pa, Soc, Chu Prong, Co ALuoi, and Lung Pu are priority breeds for conservation, while H’mong phenotypes require urgent intervention to limit further genetic erosion. Practical recommendations include the immediate integration of genomic tools such as optimum contribution selection, genome resource banking, and targeted community-based breeding programs into national conservation strategies, alongside stronger policy frameworks, infrastructure investment, and farmer capacity building. Active community engagement is essential to ensure that conservation efforts are aligned with local livelihoods and cultural values. Future research should prioritize low-coverage whole-genome resequencing to identify functional variants associated with disease resistance, heat tolerance, and feed efficiency, as well as longitudinal studies evaluating genomic selection in smallholder systems. They consequently will help to conserve indigenous pig genetic resources for food security, climate resilience, and rural development in the future.

## Figures and Tables

**Table 1:** Economic contributions of indigenous Vietnamese pig breeds.

Dimension	Major Contributions
Direct farm-level economic value	Significant share of upland household income; Low-input, low-risk production asset; Better benefit–cost ratio under extensive systems; Efficient use of household by-products; High resilience to local diseases.
Price premiums and niche markets	Strong consumer preference for taste, quality, and safety; Higher farm-gate and restaurant prices; Stable demand from specialty restaurants Incentive to conserve purebred lines.
Value-chain and rural development benefits	Supports specialized upland-lowland value chains; Creates employment for traders, transporters, and processors; Strengthens long-term trading relationships; Enables regional branding and tourism.
Risk management, resilience and option value	Acts as production “insurance” for smallholders; Tolerant to local diseases and harsh environments; Lower feed requirements reduce vulnerability to price shocks; Preserves genetic traits valuable for future breeding.
National and strategic economic benefits	Enhances food system diversity and resilience; Supports cultural heritage and national branding; Potential for premium domestic and export markets; Aligns conservation with long-term economic development.

**Table 2:** Geographic distributions and the major characteristics of indigenous pig breeds in Vietnam.

Breed	Province	Coat Color	Body Shape/Backline	Body Size ^‡^	Teat No.	Litter Size	FAO Risk Status
Northern and Northwestern Vietnam							
Muong Lay	Dien Bien	Black	Slim/Straight	Small	13.0	6–10	Endangered
Muong Te	Lai Chau	Jet black	Slim/Drooping	Small	10–12	7–8	At risk
Den (Lao Cai)	Lao Cai	Black	Slim/Drooping	Small	12–14	5–8	At risk
Muong Khuong	Lao Cai	Black + white spots	Slim/Drooping	Medium	12–14	4–6	At risk
Hung	Ha Giang	Black and white	Swayback/Drooping	Small	12–14	7–10	At risk
Lung Pu	Ha Giang	Black + white spots	Non-saggy	Medium	10–12	8–10	At risk
Tap Na	Cao Bang	Black	Slim/Drooping	Small	12–14	6–8	At risk
Ha Lang	Cao Bang	Black and white	Swayback/Drooping	Medium	12.5	8–10	At risk
Huong	Cao Bang	White + black patches	Slim/Swayback	Medium	11.8	8	At risk
Den (Cao Bang)	Cao Bang	Black	Slim/Drooping	Small	12–14	5–8	At risk
Dong Khe Spot	Cao Bang	Spotted	Slim	Small	12	6–8	Rare
Ban (Son La)	Son La	Black (some black and white)	Slim/Straight-Swayback	Small	10–12	6–8	Normal
Ban (Dien Bien)	Dien Bien	Black	Slim/Drooping	Small	12	6–7	Normal
Ban (Lai Chau)	Lai Chau	Black	Slim/Drooping	Small	12	6–7	Normal
Ban (Yen Bai)	Yen Bai	Black and white	Slim/Drooping	Small	12	6–8	Normal
Ban (Bac Kan)	Bac Kan	Black and white	Slim/Drooping	Small	12	6–8	Normal
Lung	Phu Tho	Black	Slim/Drooping	Small	12	6–8	At risk
Man	Hoa Binh	Jet black	Slim/Short	Small	10–12	5–6	At risk
Mong Cai	Quang Ninh	Black head + white body	Swayback/Drooping	Medium	14.1	10–16	Normal
I Pig (Pot–bellied)	Nam Dinh/N. Vietnam	Black	Pot-bellied/Straight	Medium	10–12	6–8	At risk
Central Vietnam							
Meo	Nghe An	Black	Broad/Flat	Medium	12–14	4–6	At risk
Xao Va	Nghe An	Black	Slim/Drooping	Small	12	5–7	At risk
Khua	Quang Binh	Black + white spots	Slim/Straight	Small	10–12	5–7	Endangered
Van Pa	Quang Tri	Black	Slim/Drooping	Small	12	6–8	At risk
Co/A Luoi	Thua Thien Hue	Black	Slim/Concave dorsal	Small	10.0	5–6	At risk
Co (Thanh Hoa)	Thanh Hoa	Black	Slim/Concave dorsal	Small	10–11	5–7	At risk
Co (Quang Nam)	Quang Nam	Black	Slim/Concave dorsal	Small	10–11	5–7	At risk
Kieng Sat	Quang Ngai	Black	Slim/Short	Small	10–12	6–9	At risk
Southern Vietnam and Central Highlands							
Chu Prong	Gia Lai	White + dark spots	Slim/Straight	Small	10–12	7	At risk
Co (Gia Lai)	Gia Lai	Black	Slim/Concave dorsal	Small	10–11	5–7	At risk
Soc	Dak Lak	Black and white	Slim/Drooping	Small	12	6–8	At risk
Co (Binh Thuan)	Binh Thuan	Black	Slim/Straight	Small	12	7–8	At risk
O Lam	An Giang	White or black and white	Medium/Straight	Large	12.9	8–12	At risk
Ba Xuyen	Mekong Delta	Black and white	Medium	Large	12–14	8–10	Extinct

‡ Body size is a qualitative frame-size descriptor following FAO DAD-IS convention, reflecting the general adult body frame of the breed independent of sex, age, feeding system, or measurement protocol. Small = generally <60 kg; Medium = generally 60–100 kg; Large = generally >100 kg under typical village/extensive conditions. Teat No.: mean (±SD) from Ishihara et al. [23] [where available; otherwise reported as range from the breed-specific literature]. FAO Risk Status: Extinct = no purebred individuals maintained; Endangered = critically small population; At risk = declining; Normal = relatively stable. Litter size data are reported as the min–max range from the breed-specific literature; mean ± SD is provided where available from Ishihara et al. [23]. Co breeds are listed by province as they represent phenotypically and genetically distinct subpopulations sharing the same local name (Ishihara et al. [23]). Breeds marked “Rare” (Dong Khe Spot) were surveyed with very small sample sizes (n = 8), and the risk status is preliminary.

**Table 3:** Major stakeholders and their roles in the conservation of indigenous pig breeds in Vietnam.

Major Stakeholders	Role
Ministry of Agriculture and Rural Development (MARD)	Oversees national policies on agriculture and livestock, including funding and implementation of conservation programs for native pig breeds; provides subsidies for keepers of at-risk breeds and supports cryopreservation.
National Institute of Animal Sciences (NIAS)	Conducts research on animal genetics, breeding, and conservation; key partner in projects like the SATREPS cryo-bank for native pig resources. Monitors the status of animal genetic resources.
Vietnam Pig Operation (VPO)	Promotes science, technology, training, and trade in the pig industry; supports production and research for sustainable development.
Institute of Biotechnology, Vietnam Academy of Science and Technology	Conducts biotechnology research for conservation, including genetic studies and cryopreservation techniques for native pig breeds.
Vietnam Academy University of Agriculture and other Agriculture universities	Provides education, research, and extension services in agriculture; involved in projects on native pig conservation and sustainable farming.
Animal Husbandry Association of Vietnam	Advocate for livestock producers; involved in conservation of at-risk breeds, sustainable use, and policy suggestions.
World Bank	Funds livestock development projects like the Livestock Competitiveness and Food Safety Project (LIFSAP, 2010–2018, concluded) to improve small-scale production, food safety, and environmental sustainability.
International Livestock Research Institute	Provides international research and collaboration on sustainable livestock systems, including conservation of native pig breeds through studies, policies, and value chain improvements.
Food and Agriculture Organization (FAO) of the United Nations	Supports global and national efforts in animal genetic resources conservation, including funding for surveys, databases, and capacity building. aids in implementing the Global Plan of Action.
Japan International Cooperation Agency (JICA) and Japan Science and Technology Agency (JST)	Fund and support technical cooperation projects, including the SATREPS project for establishing a cryo-bank system for Vietnamese native pig resources to conserve biodiversity.

## Data Availability

No new data were created or analyzed in this study. Data sharing is not applicable to this article.
